# Preservation of Pituitary Function after Endonasal Craniopharyngioma Surgery: Case Report and Review of the Literature

**DOI:** 10.7759/cureus.305

**Published:** 2015-08-17

**Authors:** Vishaal Prabhu, Vijay K Anand, Theodore H Schwartz

**Affiliations:** 1 Neurological Surgery, University of Rochester Medical Center; 2 Otolaryngology, New York-Presbyterian/Weill Cornell Medical Center; 3 Weill Cornell Brain and Spine Center, New York-Presbyterian/Weill Cornell Medical Center

**Keywords:** craniopharyngioma, endoscopic, endonasal, hormone, pituitary, surgery, transsphenoidal

## Abstract

Craniopharyngiomas comprise approximately 3% of all intracranial tumors. Preservation of pituitary function after resection represents a significant challenge due to their location in the anterior skull base and aggressive local behavior. We report a case of a 79-year-old woman with a large suprasellar craniopharyngioma who presented with visual loss. MRI revealed a suprasellar cystic mass with mass effect on the optic chiasm and pituitary gland. Following endoscopic endonasal gross total resection of the tumor, the patient’s pituitary function returned to normal.

## Introduction

Craniopharyngiomas are histologically benign epithelial tumors that arise along the path of the craniopharyngeal duct and are derived from the epithelial remnants of Rathke’s pouch [1]. Given the anatomical location of these tumors in the anterior skull base and close relation to vital structures, including the optic chiasm, pituitary gland, hypothalamus, and anterior cerebral artery, surgical resection is a significant challenge [2].

Pituitary function is especially difficult to maintain after surgery as these tumors frequently abut or strongly adhere to the pituitary gland [2]. Preservation of the infundibulum and pituitary gland often results in subtotal resection due to tumor infiltration. Thus, pituitary function is often sacrificed during surgery in order to achieve gross total resection (GTR). Here, we present a unique case of craniopharyngioma, in which the pituitary function was able to be preserved following GTR through endoscopic endonasal surgery. 

## Case presentation

**Figure 1:**
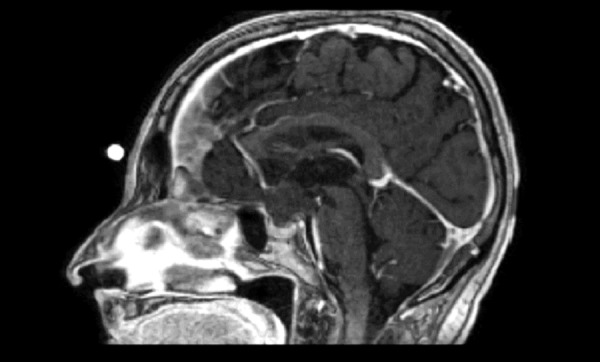
Preoperative sagittal T1 MRI with contrast shows a suprasellar solid-cystic mass compressing the optic chiasm

**Figure 2:**
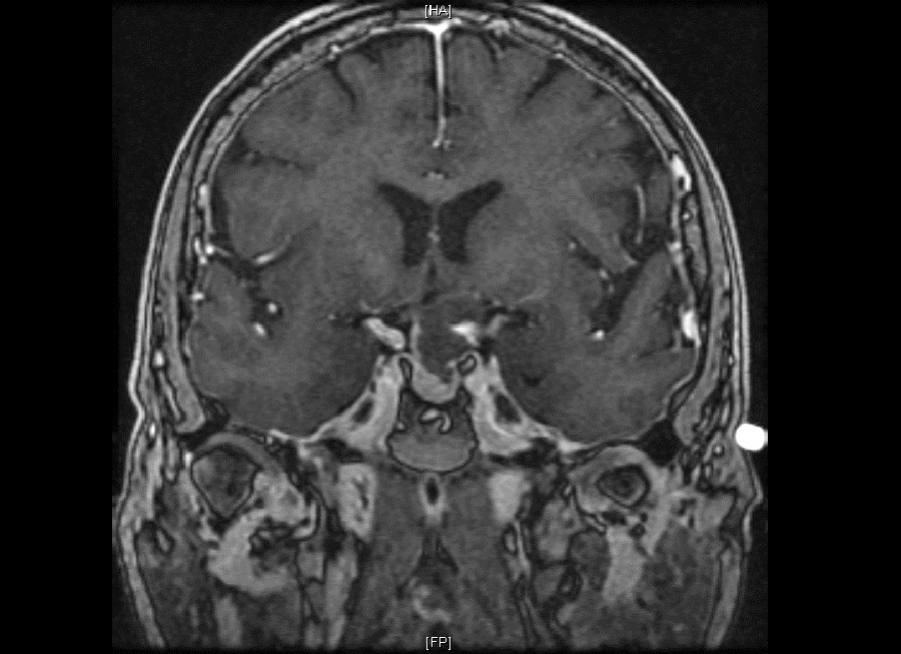
Preoperative coronal T1 MRI with contrast shows a suprasellar solid-cystic mass compressing the optic chiasm

A 79-year-old female presented with a complaint of visual disturbances over the past eight months. Signed informed patient consent was obtained prior to treatment. Upon examination, the patient was found to have a visual loss in her left eye, normal endocrine function, and intact mental status. MRI with the injection of contrast media revealed a lobulated cystic lesion centered in the suprasellar cistern measuring 2.2 x 1.6 x 2.0 cm (AP x transverse x CC). The mass demonstrated broad vascular contact with the circle of Willis as well as a mass effect on the optic chiasm, left optic nerve, and pituitary gland (Figures 1-2). CT revealed several small foci of calcification.

**Figure 3:**
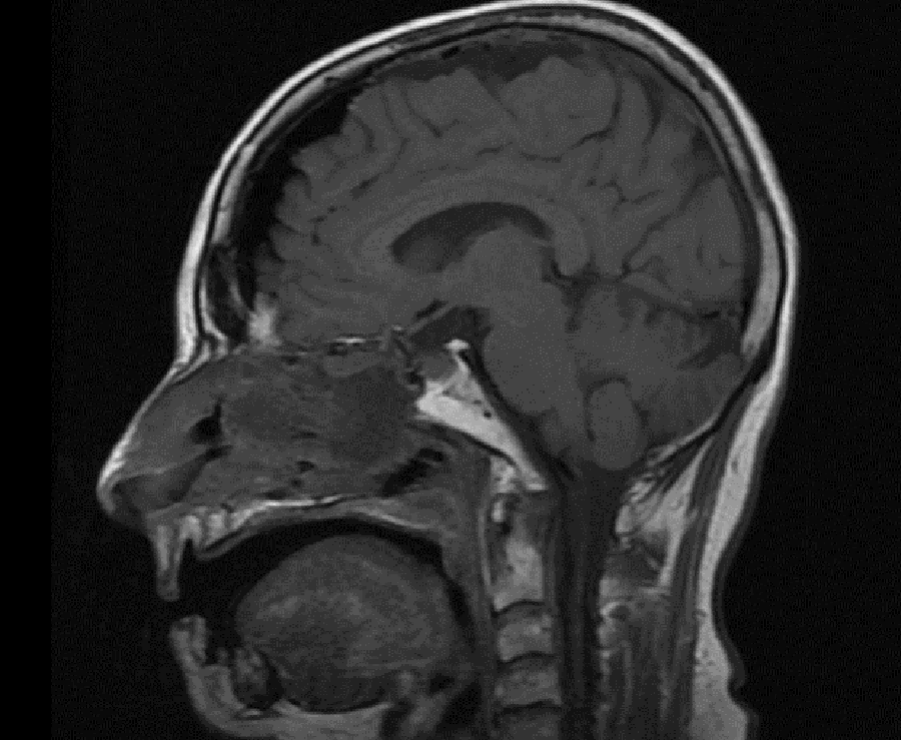
Postoperative sagittal T1 MRI shows gross total resection of the tumor

**Figure 4:**
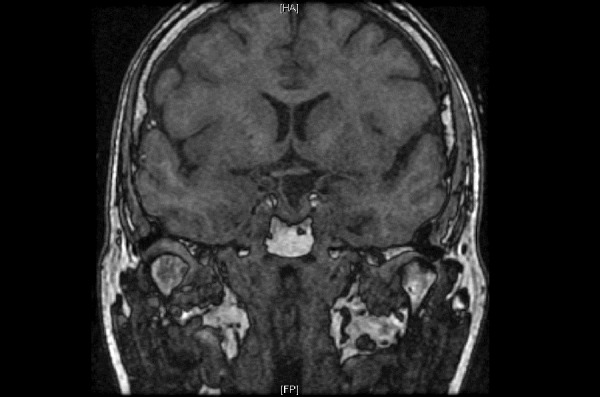
Postoperative coronal T1 MRI shows gross total resection of the tumor

The patient underwent endoscopic endonasal transsphenoidal resection of the tumor using a transtuberculum approach [2-4]. The tumor was identified and removed from the anterior skull base with no evidence of residual tumor. A gasket seal closure was used, covered by nasoseptal flap [5-7]. A lumbar drain was left in place for 24 hours. Postoperative MRI confirmed gross total resection of the tumor (Figures 3-4). The diagnosis from surgical pathology was craniopharyngioma, WHO Grade 1.

The patient did not have any complications from the procedure and was found not to have diabetes insipidus postoperatively. She did not require hormone replacement and was cleared for discharge home two days after undergoing surgery. She also reported subjective improvement in her vision.

## Discussion

Craniopharyngiomas are histologically benign epithelial tumors that arise along the path of the craniopharyngeal duct and are derived from the epithelial remnants of Rathke’s pouch [1]. Comprising approximately 3% of all intracranial tumors, craniopharyngiomas may be cystic, solid, or both and have two major pathological subtypes, namely, the adamantinomatous and squamous-papillary varieties [8-10]. Common presenting symptoms include hypopituitarism, cognitive dysfunction, and visual impairment due to mass effect [9].

Treatment of craniopharyngiomas is traditionally centered on gross total resection (GTR), although recent evidence suggests that subtotal resection and adjuvant therapy may also provide comparable control of the tumor [11]. Given the anatomical location of these tumors in the anterior skull base and close relation to vital structures, including the optic chiasm, pituitary gland, hypothalamus, and anterior cerebral artery, surgical resection is a significant challenge [2].

Pituitary function is especially difficult to maintain after surgery as these tumors frequently abut or strongly adhere to the pituitary gland [2]. Preservation of the infundibulum and pituitary gland often results in subtotal resection due to tumor infiltration. In this case, her pituitary function was able to be preserved after gross total resection through endoscopic endonasal surgery.

The endoscopic endonasal approach to the resection of craniopharyngiomas has been recently developed as an alternative to the open transcranial approach. A review of the literature suggests that the endoscopic endonasal approach may result in a greater rate of pituitary function preservation. Higher rates of pituitary function preservation may arise from improved visualization of the stalk and superior hypophyseal arteries.

A 2012 meta-analysis comparing the approaches found that the rates of permanent diabetes insipidus and hypopituitarism were 54.8% and 48.1%, respectively, in the open transcranial cohort versus 27.7% and 47.1%, respectively, in the endoscopic endonasal cohort. It also found that the endoscopic endonasal cohort had a greater rate of GTR and improved visual outcome compared to the open transcranial cohort and concluded that that endoscopic endonasal approach is a safe and effective alternative for the treatment of craniopharyngiomas [12].

In recent publications looking at outcomes of the endoscopic endonasal approach, new onset diabetes insipidus was reported in 42-48% of patients and new onset hypopituitarism was reported in 38-58% [13-15].^ ^In recent publications looking at outcomes of the open transcranial approach, new onset diabetes insipidus was reported in 66.7-93.2% of patients, new onset ACTH deficiency was reported in 56.5-83.8%, and new onset thyroid insufficiency was reported in 30.8-83.8% [16-17].

## Conclusions

Preservation of pituitary function after resection of craniopharyngioma is a significant challenge due to the location of the tumor and its frequent adherence to the pituitary gland. Endoscopic endonasal surgery can be an effective approach to gross total resection and may lead to higher rates of pituitary function preservation compared with transcranial surgeries.

## References

[1] Karavitaki N, Cudlip S, Adams BT, Wass JAH (2006). Craniopharyngiomas. Endocrine Rev.

[2] Leng LZ, Anand VK, Schwartz TH (2011). Endoscopic management of craniopharyngiomas. Operative Techniques in Otolaryngology.

[3] Conger AR, Lucas J, Zada G, Schwartz TH, Cohen-Gadol AA (2014). Endoscopic extended transsphenoidal resection of craniopharyngiomas: nuances of neurosurgical technique. Neurosurg Focus.

[4] Schwartz TH, Fraser JF, Brown S, Tabaee A, Kacker A, Anand VK (2008). Endoscopic cranial base surgery: classification of operative approaches. Neurosurgery.

[5] Leng LZ, Brown S, Anand VK, Schwartz TH (2008). “Gasket-seal” watertight closure in minimal-access endoscopic cranial base surgery. Neurosurgery.

[6] McCoul ED, Anand VK, Singh A, Nyquist GG, Schaberg MR, Schwartz TH (2014). Long-term effectiveness of a reconstructive protocol using the nasoseptal flap after endoscopic skull base surgery. World Neurosurg.

[7] Patel KS, Komotar RJ, Szentirmai O, Moussazadeh N, Raper DM, Starke RM, Anand VK, Schwartz TH (2013). Case-specific protocol to reduce cerebrospinal fluid leakage after endonasal endoscopic surgery. J Neurosurg.

[8] Bunin GR, Surawicz TS, Witman PA, Preston-Martin S, Davis F, Bruner JM (1998). The descriptive epidemiology of craniopharyngioma. J Neurosurg.

[9] Jane JA Jr, Laws ER (2006). Craniopharyngioma. Pituitary.

[10] Zada G, Lin N, Ojerholm E, Ramkissoon S, Laws ER (2010). Craniopharyngioma and other cystic epithelial lesions of the sellar region: a review of clinical, imaging, and histopathological relationships. Neurosurg Focus.

[11] Yang I, Sughrue ME, Rutkowski MJ, Kaur R, Ivan ME, Aranda D, Barani IJ, Parsa AT (2010). Craniopharyngioma: a comparison of tumor control with various treatment strategies. Neurosurg Focus.

[12] Komotar RJ, Starke RM, Raper DM, Anand VK, Schwartz TH (2012). Endoscopic endonasal compared with microscopic transsphenoidal and open transcranial resection of craniopharyngiomas. World Neurosurg.

[13] Leng LZ, Anand VK, Schwartz TH (2012). Endoscopic, endonasal resection of craniopharyngiomas: analysis of outcome including extent of resection, cerebrospinal fluid leak, return to preoperative productivity, and body mass index. Neurosurgery.

[14] Koutourousiou M, Gardner PA, Fernandez-Miranda JC, Tyler-Kabara EC, Wang EW, Snyderman CH (2013). Endoscopic endonasal surgery for craniopharyngiomas: surgical outcome in 64 patients. J Neurosurg.

[15] Cavallo LM, Frank G, Cappabianca P, Solari D, Mazzatenta D, Villa A, Zoli M, D'Enza AI, Esposito F, Pasquini E (2014). The endoscopic endonasal approach for the management of craniopharyngiomas: a series of 103 patients. J Neurosurg.

[16] Mortini P, Losa M, Pozzobon G, Barzaghi R, Riva M, Acerno S, Angius D, Weber G, Chiumello G, Giovanelli M (2011). Neurosurgical treatment of craniopharyngioma in adults and children: early and long-term results in a large case series. J Neurosurg.

[17] Hofmann BM, Hollig A, Strauss C, Buslei R, Buchfelder M, Fahlbusch R (2012). Results after treatment of craniopharyngiomas: further experiences with 73 patients since 1997. J Neurosurg.

